# Training of physical examination techniques in video conferences

**DOI:** 10.3205/zma001402

**Published:** 2021-01-28

**Authors:** Iris Schleicher, Leif Davids, Niels Latta, Anja Franziska Kreiß, Joachim Kreuder

**Affiliations:** 1 Justus-Liebig-Universität Gießen, Institut für primärärztliche Versorgung und hausärztliche Medizin, Gießen, Germany

**Keywords:** practical skills, medical teaching, online teaching

## Abstract

**Objective: **The COVID-19 pandemic also called for the teaching of practical skills to develop teaching formats outside of classroom teaching.

**Methods: **Selected physical examination techniques (musculoskeletal system, neurological system) were taught via video conference using a modified Peyton method. The core element was the mutual, real demonstration of the respective skill by student tutor and student with immediate possible correction.

**Results: **The IT requirements turned out to be sufficient, direct feedback from tutors and students was positive.

**Conclusion: **Whether this method can be a substitute for classroom courses must be evaluated in more extensive studies.

## Zusammenfassung

**Zielsetzung: **Die COVID-19-Pandemie forderte auch für den Unterricht von praktischen Fertigkeiten die Entwicklung von Lehrformaten außerhalb der Präsenzlehre.

**Methodik: **Per Videokonferenz wurden ausgewählte körperliche Untersuchungstechniken (Bewegungsapparat, neurologisches System) mit einer modifizierten Peyton-Methode gelehrt. Kernelement war die wechselseitige, reale Demonstration der jeweiligen Fertigkeit durch studentische Tutor*in und Student*in mit unmittelbar möglicher Korrektur.

**Ergebnisse: **Die informationstechnischen Voraussetzungen erwiesen sich als ausreichend, das direkte Feedback der Tutor*innen und Studierenden*innen war positiv.

**Schlussfolgerung: **Ob diese Methode einen Ersatz für Präsenzkurse darstellen kann, muss in umfangreicheren Studien evaluiert werden.

## 1. Introduction

Since 2009, at the Department of Medicine of the Justus Liebig University Giessen, basic practical skills have been successfully taught in small groups of 4 and occasionally 6 students by student peers in accordance with the Peyton method [1]. 

Due to the COVID-19 pandemic we were forced to use digital teaching formats at short notice during summer semester 2020, which is especially challenging for practical courses [2], [3]. For selected courses such as “Examination of the musculoskeletal system” and “Neurological examination” we developed an online course, which seemed to make it possible to demonstrate and correct these skills via video conferencing. The availability of student tutors was limited due to the pandemic, as many students did not stay at their study location. The course also required an expansion of information technology requirements.

## 2. Methods

For the online courses “Examination of the musculoskeletal system” and “Neurological examination”, 2 tutors for 4 course participants were provided, so that on one hand the tutor could demonstrate the examination technique at one person and on the other hand each tutor could concentrate on only 2 students when correcting the learned technique. As in the previous classroom courses, the tutors were trained by a senior doctor beforehand. Registration for the course was made via the learning platform “k-med”. For the video courses the program “Cisco Webex Meetings” was used. The link to each course and the registration data at k-med were uploaded for the respective course. The students could then take part at the course staying home and using a laptop/computer with camera. The course participants should demonstrate the various examinations on a person from their home area.

The online course “Examination of the musculoskeletal system” consisted of two consecutive courses of one hour each with examination of the shoulder and knee joint (part 1) and spine, hip joint and ankle joint (part 2). The course “Neurological Examination” of one hour duration included the basic neurological examination. 

Prerequisite for participation was the prior study of selected textbook chapters and instructional videos from the book “Heidelberg Standard Examinations”. 

At the beginning, the students were asked to switch on their camera, due to the background noise, the sound should remain switched off except for questions. 

Anatomical features and the structured examination were first explained by the tutors using models or display boards.

Subsequently, the tutors demonstrated the examination in real life on the 2^nd^ tutor. The students could observe this on the screen and ask questions at any time. Afterwards, the students were asked to demonstrate the examination on the person they had previously chosen and were observed and corrected synchronously by the tutors. One tutor was responsible for 2 students.

In between and at the end of the examinations there was enough time for questions or renewed demonstration of certain examinations and feedback.

At the end of the course, a link to an online evaluation form was sent to the students.

## 3. Results

In the examination course “musculoskeletal system” part 1 and 2 with 4 participants and 2 student tutors on 2 consecutive days 200 students participated, in the course “neurological examination” 212 students participated. 

Despite great fears at the beginning with regard to possible mutual technical problems or difficulties in observing the examination technique shown on a screen with sufficient accuracy, all courses were carried out smoothly. The tutors were able to demonstrate, observe and correct the respective examination well. 

The feedback from the students and tutors at the end of the course was very positive. Unfortunately, however, the evaluation forms that were sent later online were returned only sparsely, so that a statistical evaluation was not possible. Therefore we could only use individual comments as written feedback.

Some participants in the musculoskeletal system examination course had problems finding another person for demonstration of the skill, but rated the course as good overall, although not as instructive as a classroom course. In the neurological examination course, some students felt that they had not learned much new in terms of their practical skills and their clinical significance. There was also criticism that the skills could not be demonstrated and corrected sufficiently well online.

## 4. Discussion

Due to the COVID-19 pandemic, medical faculties have been forced to use digital teaching methods [2]. In addition to online seminars, provision of videos and learning content on learning platforms, we were looking for ways to demonstrate and correct practical skills online. The courses presented involved a not inconsiderable amount of organization, technical requirements and availability of student tutors. Even though we do not believe, based on our experience, that this course can completely replace classroom teaching with practical exercises, we think that we were able to teach selected physical examination techniques that can be easily corrected by observation alone [3]. Furthermore, due to the predominantly synchronous online format, the course concept presented here goes far beyond other published virtual adaptations of the Peyton method [4]. As a consequence of the poor response rate to the evaluation, we will have an online evaluation form filled out directly after the course in the forthcoming semester. It is certainly worth considering whether a final demonstration of a skill with correction using a checklist (like an OSCE) would increase the value of the course as well as the students’ preparation and cooperation. Furthermore, it is of decisive importance to exchange experiences with the new formats that are new to all of us in this consequence and, if necessary, to be able to maintain them as a well transferable course concept for selected topics even beyond the pandemic period [5], [6].

## Competing interests

The authors declare that they have no competing interests. 
